# Chimera: a block-based neural architecture search framework for event-based object detection

**DOI:** 10.3389/frai.2025.1644889

**Published:** 2025-10-01

**Authors:** Diego A. Silva, Ahmed Elsheikh, Kamilya Smagulova, Mohammed E. Fouda, Ahmed M. Eltawil

**Affiliations:** ^1^Communication and Computing Systems Lab, Computer, Electrical and Mathematical Sciences and Engineering Division, King Abdullah University of Science and Technology, Thuwal, Saudi Arabia; ^2^Mathematics and Engineering Physics Department, Faculty of Engineering, Cairo University, Giza, Egypt; ^3^Compumacy for Artificial Intelligence Solutions, Cairo, Egypt

**Keywords:** neural architecture search, event-based cameras, object detection, neuromorphic datasets, Zero-Shot NAS, hybrid neural networks

## Abstract

Event-based cameras are sensors inspired by the human eye, offering advantages such as high-speed robustness and low power consumption. Established deep learning techniques have proven effective in processing event data, but there remains a significant space of possibilities that could be further explored to maximize the potential of such combinations. In this context, Chimera is a Block-Based Neural Architecture Search (NAS) framework specifically designed for Event-Based Object Detection, aiming to systematically adapt RGB-domain processing methods to the event domain. The Chimera design space is constructed from various macroblocks, including attention blocks, convolutions, State Space Models, and MLP-mixer-based architectures, providing a valuable trade-off between local and global processing capabilities, as well as varying levels of complexity. Results on Prophesee's GEN1 dataset demonstrated state-of-the-art mean Average Precision (mAP) while reducing the number of parameters by 1.6 × and achieving a 2.1 × speed-up. The project is available at: https://github.com/silvada95/Chimera.

## 1 Introduction

Object detection is a critical task in computer vision that involves identifying objects and determining their locations within an image. This capability is essential for various real-world applications, including autonomous driving (Michaelis et al., 2019), robotics (Xu et al., 2022), and surveillance (Jha et al., 2021). Traditionally, these applications rely on data from RGB cameras, which provide a continuous stream of high-resolution images (Liu et al., 2020). Recently, event-based cameras were introduced as a new sensing paradigm, inspired by the human eye's functioning (Lichtsteiner et al., 2008). Unlike traditional cameras, pixels in event-based sensors generate outputs independently only when changes occur in the scene, leading to a spatio-temporal stream of events in response to brightness variations. Event-based sensors offer several advantages over RGB cameras, such as microsecond-range latency, a High Dynamic Range (HDR) exceeding 120 dB, power consumption in the milliwatt range, and potential memory savings by discarding redundant information (Gallego et al., 2022).

Among the various techniques developed for object detection using RGB input, deep learning algorithms, particularly the You-Only Look-Once (YOLO) family and transformer-based detectors—have achieved significant success (Liu et al., 2020). Various YOLO versions were introduced, enhancing its speed and accuracy while maintaining minimal trainable parameters (Terven et al., 2023). There is a notable correlation between the success of deep learning methods in RGB applications and their performance in the event-based domain, as seen with convolutional networks (Perot et al., 2020; Li et al., 2022a; Silva et al., 2025), and transformer-based networks (Gehrig and Scaramuzza, 2023; Peng et al., 2024, 2023b; Zubic et al., 2024). Many of these networks are designed monolithically, meaning they consist of repeated layers of the same blocks.

Additionally, in conventional computer vision, integrating various architectural blocks into a single hybrid network has shown significant benefits. Specifically, employing convolutions in the trunk layers and transformers in later stages has proven effective for balancing local and global contextual processing while managing computational complexity across diverse feature sizes (Hatamizadeh et al., 2023; Tu et al., 2022; Chen et al., 2022; Li et al., 2022b). Some studies have also explored combinations such as convolutional layers with MLP-Mixers (Li et al., 2023) and State-Space Models with Transformers (Hatamizadeh and Kautz, 2024). Typically, the choice of these combinations is influenced by researchers prior knowledge and experience. However, this process can be automated through the use of Neural Architecture Search (NAS) frameworks (Ren et al., 2021). In this context, Zero-Shot NAS (ZS-NAS) emerges as a promising research avenue by providing proxies that can evaluate the potential of different neural network configurations without the need for extensive training (Li et al., 2024).

**Figure 1:**
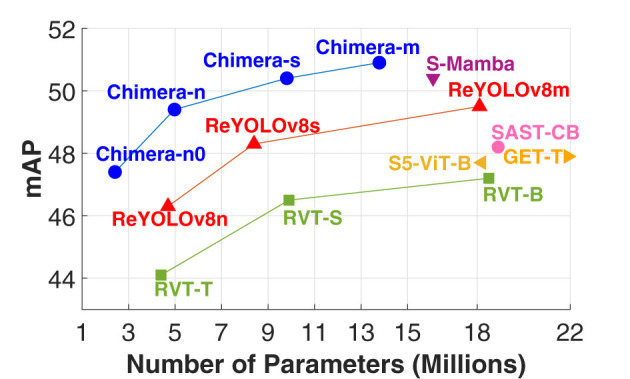
Graph showing mAP versus the number of parameters in millions for various models. Chimera models, in blue, and ReYolOv8 models, in red, show varied mAP values between 44 and 52 across different parameter counts. Other models, such as S-Mamba, S5-ViT-B, RVT, and SAST-CB, display lower mAP values with higher parameters. Performance of Chimera for the prophesee's GEN1 dataset.

The use of ZS-NAS makes the process more accessible and feasible. Instead of requiring full training—which often leads to high computational costs and sustainability concerns (Patterson et al., 2021)—ZS-NAS utilizes proxy metrics for candidate evaluation, offering improved scalability, speed, cost-efficiency, and sustainability. Inspired by the good performance reported for hybrid models in the frame-based literature, as well as the possibilities of automatizing their design process leveraging ZS-NAS, this work introduces a scalable, two-stage Neural Architecture Search (NAS) framework named Chimera, specifically designed to identify heterogeneous architectures for event-based applications through the integration of well-known building blocks and proxies adopted in the literature. This framework was benchmarked using the PeDRo dataset, analyzing various event encodings and model configurations. The resulting architectures demonstrated strong generalization capabilities, achieving state-of-the-art results on Prophesee's GEN1 dataset while being faster and requiring fewer parameters than the top-performing model from the literature. Figure 1 highlights the benefits of the models developed in this work when compared to the literature.

## 2 Related works

### 2.1 Event-based object detection

Currently, there are various neural architectures available for vision tasks. Existing event-based object detectors can be divided into two primary categories based on their processing approach: sparse models and dense models. Sparse models process input event streams asynchronously and include techniques like Graph Neural Networks (GNN) (Schaefer et al., 2022; Sun and Ji, 2023; Gehrig and Scaramuzza, 2024) and Spiking Neural Networks (SNN) (Kugele et al., 2021; Cordone et al., 2022; Su et al., 2023; Zhang et al., 2023; Wang et al., 2023; Bulzomi et al., 2023; Fan et al., 2024; Wang et al., 2024). In contrast, dense models convert event streams into an intermediate format suitable for neural networks that process image-like features. The most common and effective configurations for dense neural networks are built using convolutional layers (Perot et al., 2020; Li et al., 2022a; Peng et al., 2023a; Liu et al., 2023; Silva et al., 2025), as well as self-attention blocks and their variants (Gehrig and Scaramuzza, 2023; Peng et al., 2024, 2023b; Zubić et al., 2023; Zubic et al., 2024). Additionally, several architectures integrate Recurrent Neural Networks (RNNs) to enhance temporal modeling (Perot et al., 2020; Gehrig and Scaramuzza, 2023; Li et al., 2022a). Notably, State Space Models (SSM) (Zubic et al., 2024; Yang et al., 2025), and Hierarchical Memory Networks (HMNet) (Hamaguchi et al., 2023) are also implemented in this context. Although significant progress has been done on sparse models, there is still a gap in performance between them and the dense approaches, which motivates adopting the latter in this work.

### 2.2 Hybrid neural networks

Combining diverse blocks into a hybrid architecture and leveraging their complementary features can enhance performance while achieving a balanced trade-off between computational complexity and global/local modeling (Li et al., 2023; Gu and Dao, 2023). For example, transformer-based models are recognized for their state-of-the-art accuracy in vision applications (Vaswani, 2017), but their high computational complexity can make processing high-resolution images challenging. To mitigate this issue, it is common practice to employ convolutional layers in the initial stages for input downsampling, followed by transformer-based blocks as the resolution decreases (Hassani et al., 2021). This approach helps to maintain a balance between local and global feature modeling throughout the network (Chen et al., 2022; Tu et al., 2022; Hatamizadeh et al., 2023; Li et al., 2022b).

Similarly, convolutional layers have been used with MLP-Mixers to accommodate arbitrary input resolutions while reducing computational complexity (Li et al., 2023). In EfficientVMamba, an integration of convolutional blocks with State Space Models (SSM) was implemented, but unlike previous approaches, the SSM blocks were positioned in the early stages of the network to maximize global feature capture, with convolutional layers placed in the later stages (Pei et al., 2024). Conversely, MambaVision (Hatamizadeh and Kautz, 2024) employs convolutional layers at higher resolution layers while incorporating a mixer block that alternates between Mamba (Gu and Dao, 2023), an SSM block, and self-attention (Vaswani, 2017). Other methodologies explore modifications of convolutional blocks with self-attention (Srinivas et al., 2021), the reverse (Chen et al., 2021a; Xu et al., 2021; Chen et al., 2021b; Hatamizadeh et al., 2023), and even the creation of novel blocks that combine both paradigms (Chen et al., 2021b; Tu et al., 2022; Chen et al., 2022; Wu et al., 2021).

### 2.3 Zero-Shot NAS

Neural Architectural Search (NAS) was developed to automate the process of finding the structure and design of neural networks considering the given constraints to improve performance In this work, the preference is given to the Zero-Shot NAS, which eliminates the need for training neural networks and, therefore, improves cost and time efficiency (Lin et al., 2021). Moreover, it offers high scalability and can be optimized for specific metrics using zero-shot proxies. The proxies are developed based on theoretical and empirical analysis of deep neural networks, incorporating factors such as topology, initialization, gradient propagation, etc. Understanding how they impact the overall performance enhances interpretability and predictions.

The implementation of Zero-Shot NAS requires identifying a design space of candidates F and selection of proxy metrics. As a result, the framework evaluates the candidate architectures, ranks them according to the estimated proxy scores, and selects the top architectures.

## 3 Methodology

In this work, we proposed a Neural Architecture Search (NAS) algorithm designed to identify optimal hybrid neural networks, with focus on event-based applications. The resulting architectures interleave blocks from different paradigms—such as convolutions and transformers—across various stages of the network. The overall topology is inspired by Recurrent YOLOv8 (ReYOLOv8) (Silva et al., 2025), featuring a recurrent backbone module for input feature extraction, along with the multi-scale feature fusion and detection heads from the original YOLOv8 model (Jocher et al., 2023).

### 3.1 Event encodings

The input to the framework is assumed to be an event stream. Each event within an event stream arises from changes in the brightness and can be represented as a sequence *e*_*k*_ = (*x*_*k*_, *y*_*k*_, *t*_*k*_, *p*_*k*_) for *k* = 1, 2, …, *N*, where (*x, y*) denotes the pixel location, *t* indicates the timestamp and *p* reflects the polarity. A simple method for transforming an event stream into a dense, grid-like format to be suitable for later neural network processing involves stacking the events in various configurations. The formats analyzed in this work were Volume of Ternary Event Images (VTEI) (Silva et al., 2025), which was associated with good performance on ReYOLOv8, Stacked Histograms (SHIST), adopted on RVT (Gehrig and Scaramuzza, 2023), and some subsequent works (Peng et al., 2024, 2023b; Zubic et al., 2024), Mixed-Density Event Stacks (Nam et al., 2022), which proposes a different way of creating temporal bins and showed to be successful on depth estimation application, and Temporal Active Focus (TAF) (Liu et al., 2023), a First-In First-Out (FIFO)-based approach.

### 3.2 Chimera network organization

**Figure 2:**
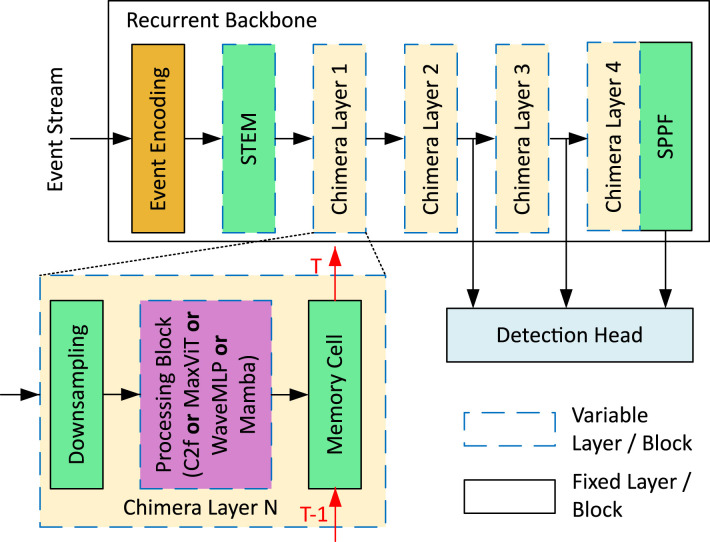
Flowchart illustrating a multi-step process. It starts with an orange rectangle leading to several green and beige rectangles, representing different stages with arrows showing progression. A section is zoomed in to show more detail, highlighting interactions within a purple rectangle. A key at the bottom right uses various shapes, patterns, and colors to denote different components. Structure of the Chimera network.

Figure 2 displays the fundamental architecture of Chimera's recurrent backbone, which consists of seven layers. It begins with an Event Encoding block and a 3 × 3 downsampling convolutional STEM layer. The subsequent four layers are called Chimera layers, each having an identical structure but varying compositions. These four layers comprise downsampling, processing, and a memory cell. The downsampling components resemble the STEM layer, while the memory cell is a fixed structure based on Convolutional Long-Short Term Memory (ConvLSTM) blocks (Shi et al., 2015). The ConvLSTM is modeled after a standard LSTM (Hochreiter and Jürgen, 1997) but adapted to process spatial features, as done in Recurrent Vision Transformer (RVT) (Gehrig and Scaramuzza, 2023) and ReYOLOv8 (Silva et al., 2025). This memory cell performs spatiotemporal modeling between the current and previous feature maps. The processing block can utilize any option available in Chimera's component library, and the choice of block for each Chimera Layer is made independently from the others. The final layer of the recurrent backbone is a fixed Spatial Pyramid Pooling Fast (SPPF) (He et al., 2015) block, stacked to Chimera Layer 4 and is inherited from YOLOV8 (Jocher et al., 2023). Detection within the Chimera framework also utilizes the multi-scale YOLOV8 detection head, similarly to Silva et al. (2025).

### 3.3 Building blocks

The library supporting Chimera comprises various building blocks. This section will provide a brief overview of each component.

#### 3.3.1 C2f block

**Figure 3:**
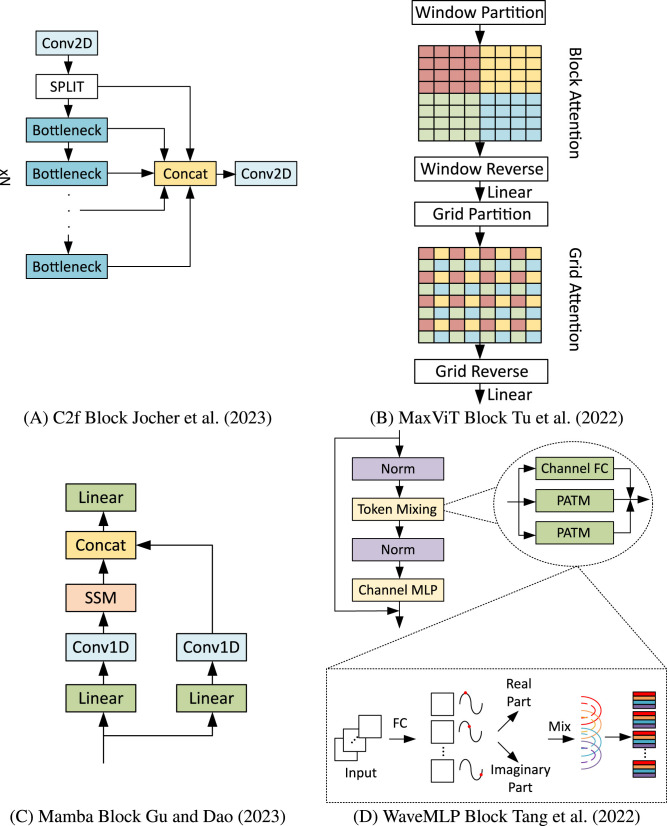
Diagram with four labeled sections. (A) shows a flowchart with interconnected rectangles representing data processing steps. (B) illustrates a grid with colored squares and arrows indicating transformations. (C) depicts a vertical sequence of rectangles linked by arrows, indicating a process flow. (D) contains two detailed insets with rectangles and a series of arrows, demonstrating complex data manipulation steps. Building blocks adopted in the Chimera framework. **(A)** C2f block (Jocher et al., 2023). **(B)** MaxViT block (Tu et al., 2022). **(C)** Mamba block (Gu and Dao, 2023). **(D)** WaveMLP block (Tang et al., 2022).

The well-recognized capability of Convolutional Neural Networks (CNNs) to extract features has significantly transformed various computer vision tasks (Krizhevsky et al., 2012). For example, YOLOV8, which serves as the foundation of the Chimera framework, is composed of backbone, neck, and head blocks made entirely of convolutions, like the downsampling convolutions and the C2f blocks adopted for finer feature extraction (Jocher et al., 2023). Hence, all those convolutions will be kept in Chimera, and the C2f blocks will be adopted as one of the possible choices for the Chimera Layers. A block diagram of C2f can be seen in Figure 3A.

#### 3.3.2 MaxViT block

Transformers are highly powerful in modeling global context information due to the presence of self-attention operations (Vaswani, 2017). However, this operation has quadratic complexity concerning the input size, which incurs computational burdens. In this context, Multi-axis Vision Transformer (MaxViT) (Tu et al., 2022), a variation of self-attention with reduced computational complexity, was included in the Chimera library. Remarking that this block was already successfully adopted in the event domain (Gehrig and Scaramuzza, 2023) is worthy. In the Chimera framework, this block is instantiated only in terms of input and output channels. The remaining parameters are the same as those used in RVT, including the decision not to stack such blocks. Figure 3B depicts its main operations.

#### 3.3.3 Mamba block

Grounded in continuous-time linear systems, these models have recently gained prominence for their efficiency in parallel processing. Various models adhering to this principle have emerged, mainly differing in matrix representations. The Mamba block, which is included in the library, has attracted significant attention recently, both in the context of Large Language Models (LLMs) (Gu and Dao, 2023) and in the vision domain (Hatamizadeh and Kautz, 2024), including even applications on the event-based domain (Yang et al., 2025). In the original implementation, the Mamba block alternates between a State Space Model (SSM) and Self-Attention mechanisms within the same stage. However, it was decided to retain only the SSM block for the Chimera framework, as MaxViT already incorporates Self-Attention. This approach enables us to evaluate the effects of the Mamba block in a standalone manner within a specific stage. Details of its structure are presented in Figure 3C.

#### 3.3.4 WaveMLP

Multilayer Perceptron (MLP)-Mixers model local and global relationships through channel and token mixing (Tolstikhin et al., 2021). Token mixing captures spatial information, while channel mixing focuses on feature information. WaveMLP is an MLP-Mixer that treats tokens as waves, incorporating amplitude and phase information and introducing a Phase-Aware Token Mixing module (PATM) (Tang et al., 2022). Due to its flexibility and reported performance, WaveMLP was included in the Chimera library. A simplified diagram can be seen in Figure 3D.

#### 3.3.5 YOLOv8 detection head and PANET

**Figure 4:**
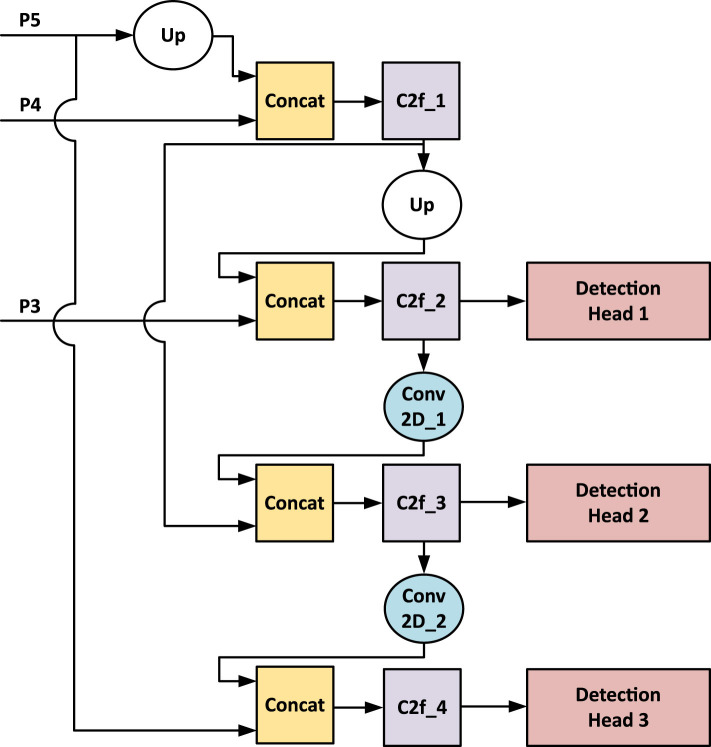
Flowchart of a neural network architecture. It starts with inputs P5, P4, and P3, with P5 undergoing an upward process labeled “Up." These merge at “Concat" blocks, followed by “C2f" and “Conv 2D" processing units, leading to three detection heads labeled Detection Head 1, 2, and 3. Arrow lines indicate data flow between processing units. Multi-scale feature fusion and Detection Head structures from YOLOv8.

Like other algorithms in the YOLO family, YOLOv8 comprises structures responsible for feature extraction, multi-scale feature fusion, and a detection head. As illustrated in Figure 2, the last three feature maps from the backbone are forwarded to the Detection Head, which, for simplicity in this discussion, encompasses both the multi-scale feature fusion structure and the detection heads. The multi-scale feature fusion in YOLOv8 utilizes a Path Aggregation Network (PANET) that fuses those features from the backbone and transmits them to three detection heads. Figure 4 displays both structures, with *P*5, *P*4, and *P*3 denoting the last three feature maps from the backbone. The number of channels of the PANET are fixed with respect to the choice of output channels from the STEM layer.

### 3.4 Building Chimera-NAS fitness function

In this work, Zero-Shot NAS proxies were adopted as the core mechanisms to search through the hybrid design space. Zero-Shot NAS proxies are quantities that are calculated during the networks' initialization, which enable the estimation of the test-set performance without undergoing training setups, turning the search process less resource demanding (Li et al., 2024). The fitness score adopted in the ZS-NAS search process is built from a linear combination of different proxies that are described in this section.

#### 3.4.1 ZenScore

The expressive capacity of a network refers to its ability to effectively capture complex relationships within the input data. For vanilla CNNs, it can be associated with Gaussian complexity according to the following equation:


(1) $$\phi (f)=\log E_{x,\theta }\|\nabla_{x}f(x|\theta ){\|}_{F}$$

where **x** is the input, **θ** the network parameters, and *f*(.) is the network backbone with the last feature before the Global Average Pooling (GAP) operation. The formulation from Equation 1 considers a network without Batch Normalization layers. However, this leads to problems such as overflow when applied to deep networks. The Zen-score solved this problem by introducing Batch Normalization layers and considering their variance into the score computation (Lin et al., 2021). Furthermore, to avoid adopting the backward propagation from Equation 1, they calculate the score according to the finite differential:


(2) $$\Delta =E_{x,\epsilon }\|f(x)-f(x+\alpha \epsilon ){\|}_{F}$$

where **ϵ** is a random disturbance and α is an adjust parameter for this noise. Then, the Zen-score is given by:

(3) $$\begin{array}{l} Zen\left(f\right)=\log\left(\Delta \right)+\underset{i}{\sum }\log\left(\sigma_{i}\right) \end{array}$$

where *i* refers to the index of the Batch Normalization layers, each with its respective standard deviation σ_*i*_. Originally, both **x**, **θ**, and **ϵ** were taken from a standard Gaussian Distribution (Lin et al., 2021). Also, in Chimera, *f*(.) will consider the whole backbone block, including the Spatial Pyramid Pooling-Fast (SPPF) block.

#### 3.4.2 Minimum eigenvalue of correlation (MeCo)

The Minimum Eigenvalue of Correlation (MeCo) score was developed by exploiting the similarities between multi-channel CNN layers and over-parameterized NN layers and the relationship between the generalization capacity of an NN and the minimum eigenvalue of its Pearson correlation matrix (Jiang et al., 2023). Based on that, for a *L*-layered network *f*^*i*^(**X**; **θ**), the MeCo proxy is defined as:

(4) $$\begin{array}{l} MeCo:=\overset{L}{\underset{i=1}{\sum }}\lambda_{min}\left(P\left(f^{i}\left(\text{X};\text{θ}\right)\right)\right) \end{array}$$

where λ_*min*_ is the minimum eigenvalue of the Pearson correlation matrix *P*(*f*^*i*^(**X**; **θ**)) of the *i*-th layer *f*^*i*^(**X**; **θ**) randomly initialized with parameters **θ** and evaluated over the dataset **X**.

#### 3.4.3 AZ-NAS proxies

In AZ-NAS (Lee and Ham, 2024), the authors proposed an ensemble of different proxies to develop their NAS algorithm. Firstly, they defined an expressivity index based on the Principal Component (PC) Analysis. The reasoning is that the less correlated the eigenvalues, the higher the capacity for network generalization. By performing an eigenvalue decomposition to obtain those PCs, the scores per layer are calculated as an entropy score that takes those eigenvalues as probabilities. This can be calculated as:

(5) $$\begin{array}{l} {AZ_{l}}^{expr}=\overset{c}{\underset{i=1}{\sum }}-{\tilde{\lambda }}_{l}\left(i\right)\log{\tilde{\lambda }}_{l}\left(i\right) \end{array}$$

where *c* is the number of features of the *l*-th layer and ${\tilde{\lambda }}_{l}$ the eigenvalues for that layer obtained through PC analysis. They associate the expressiveness with the isotropy of the feature space, checking whether there are dominant eigenvalues, which could result in the problem of dimensional collapse. Then, the AZ-NAS score for a whole architecture is given by:

(6) $$\begin{array}{l} {AZ}^{expr}=\overset{L}{\underset{i=1}{\sum }}{AZ_{l}}^{expr} \end{array}$$

They also proposed an auxiliary proxy to measure the progressivity of a network, which measures the capability of expanding the size of a network through the increase of its depth, represented here as *AZ*^*prog*^ (Lee and Ham, 2024). This proxy can be calculated as:

(7) $$\begin{array}{l} {AZ}^{prog}=\underset{l\in \left\{2,...,L\right\}}{\text{min}}\left({AZ_{l}}^{expr}-{AZ_{l-1}}^{expr}\right) \end{array}$$

which is the slightest difference in expressivity on the neighboring blocks. The higher this value is, the more consistent the expressivity increases from one layer to another. They also added a metric for trainability, which is based on the observation that the spectral norm of a Jacobian matrix for each layer being close to 1 is related to good propagation of gradients through a network (Lee and Ham, 2024). This index is calculated as:

(8) $$\begin{array}{l} {AZ}^{train}=\frac{1}{L-1}\overset{L}{\underset{l=2}{\sum }}\left(-\sigma_{l}-\frac{1}{\sigma_{l}}+2\right) \end{array}$$

where *L* is the number of layers and σ_*l*_ is an approximation of the spectral norm of the Jacobian matrix. Adopting σ_*l*_ and its reciprocal is meant to punish values far from 1.

#### 3.4.4 Model complexity

The complexity of a model, measured in terms of Multiply-Accumulate Operations (MACs), has been found to correlate with the model's test accuracy. As a result, this metric has also been incorporated into the analysis presented here (Li et al., 2024).

### 3.5 Chimera-NAS search algorithm

#### 3.5.1 Algorithm

The design space F of the Chimera framework is characterized by the backbone and the multi-scale feature fusion structures. As shown in Table 1, the backbone design begins by selecting the STEM layer output channels *Ch*, which is 3 × 3 convolution with stride 2. Each subsequent Chimera Layer is assigned a channel multiplier Mi, which determines its number of channels relative to preceding layers, along with a designated processing unit “Block”. A parameter “Repeats” is defined for all blocks except the MaxViT block: for the C2f block, “Repeats” is the number of bottleneck blocks inside its architecture; for the other blocks, it is the number of block instances stacked. On the other hand, the multi-scale feature fusion has a fixed structure (Figure 4), where only the output channels of its C2f blocks are adjustable. In Table 2, for each STEM output channel value *Ch*, a value Ch0 is chosen. Table 3 then shows that the output channels *Ch*0 of each PANET C2f block are determined by predefined multipliers based on the size constraint *MAX*_Params_ and the chosen Ch0. This change of multipliers related to the maximum architecture size is meant to prevent the detection head blocks from consuming most of the available resources. All channel numbers are rounded to a multiple of 8 for better resource utilization. This design space can generate approximately 111 million unique architectures.

**Table 1:** Design choices present on the Chimera library backbone.

**#**	**Layer**	**Search focus**	**Choices**
1	STEM	Output Channel *Ch*	16, 24, 32, 40, 48
2-5	Chimera Layers 1-4	Multiplier *M*_*i*_	1, 1.25, 1.33, 1.50, 1.66, 1.75, 2.00
				Block	C2f, MaxViT, Mamba, WaveMLP
				Repeats (Except MaxViT)	1, 2, 3

**Table 2:** Relationship between the width parameter of the multi-scale feature fusion block and the STEM's layer output channel.

**STEM's layer Output Channel *Ch***	**Channel adopted across the PANET, Ch0**
16	16
24	20, 24
32	28, 32
40	36, 40
48	44, 48

**Table 3:** Channel parameters of the different multi-scale feature fusion blocks according to the parameter constraint of the model.

	***MAX*_Params_ <14M**	***MAX*_Params_ >14M**
**Block**	*Ch*_*out*_	
C2f_1	8*Ch*_0_	8*Ch*_0_
C2f_2	4*Ch*_0_	4*Ch*_0_
C2f_3	8*Ch*_0_	8*Ch*_0_
C2f_4	16*Ch*_0_	12*Ch*_0_

The search algorithm within the Chimera framework proceeds in two distinct stages. In the first stage, architectures are generated and selected using an Evolutionary Algorithm, which is preferred due to its simplicity and effectiveness, as evidenced by prior results in the ZS-NAS domain (Lin et al., 2021). The Fitness Score can be computed using any of the metrics outlined in Section 3.4, or as a combination of these metrics. The optimization problem that this first stage aims to address, given a design space F, can be framed as follows:


(9) $$\begin{array}{l} \underset{f\in ℱ}{\text{max}}\;F=W\cdot ZS(f)\\ \text{s.t. }Params(f)\leq MAX_{\text{Params}} \end{array}$$

In this formulation, **ZS(*f*)**∈ℝ^1 × *N*^ represents a vector comprising a set of *N* ZS-NAS proxies calculated for the architecture *f*, weighted by the vector **W**∈ℝ^*N*×1^. The term *Params*(*f*) denotes the number of parameters within the architecture *f*, while *MAX*_Params_ is an upper limit for model sizes. The proxies' mean value and standard deviation are utilized to standardize them, allowing for representation on a unified scale.

During the second stage, the top five architectures identified by the ZS-NAS process are trained for 100 epochs. Ultimately, the architecture that achieves the highest mean Average Precision (mAP) is chosen. This second step is essential for mitigating the inherent inaccuracies associated with proxy-based methods and fine-tuning the resulting architecture.

#### 3.5.2 Chimera testbed

To evaluate the accuracy of the selected proxies in predicting performance on the test set and to determine the optimal weight vector for Equation 9, a testbed was established using a subset of architectures from the Design Space. The PeDRo dataset (Boretti et al., 2023) was chosen for this task due to its small size, serving as the basis for profiling the proxies and conducting the Chimera search. This methodology was inspired by frame-based approaches, which typically utilize smaller datasets such as CIFAR-10 or CIFAR-100 before advancing to larger datasets like ImageNet1k (Liu et al., 2018). Each model was trained for 50 epochs, effectively balancing runtime and convergence, and the test set's mean Average Precision (mAP) was recorded. This analysis involved executing 1,250 randomly generated models, incorporating heterogeneous and homogeneous compositions of all blocks from the library. Each model was trained using the VTEI, MDES, TAF, and SHIST representations, each consisting of five temporal bins, to evaluate the relationship between event encodings and different architectures. The choice to use five temporal bins follows the approach implemented in ReYOLOv8 (Silva et al., 2025), which demonstrated strong performance. Comparable values are reported in the literature; for example, RVT utilized six temporal bins (Gehrig and Scaramuzza, 2023).

Two correlation measures were employed to evaluate the effectiveness of the proxies in approximating the mAP ground truth. The first measure, Kendall's Tau, compares models' rankings based on mAP with those determined by the proxies. The second measure, Spearman's correlation, assesses the degree of monotonicity between the two variables: the proxies and the mAP (Li et al., 2024). Additionally, the mean squared error of the top 10% of mAPs relative to the mAPs sorted by each proxy was analyzed.

Regression Trees (Breiman et al., 2017) were utilized to determine the optimal weights for Equation 9, which were subsequently applied in Algorithm 1, using the mAPs from the testbed as the target values.

**Algorithm 1:** Chimera algorithm.

1: ————————- First Stage: ZS-NAS —————————–
2: Input: Population size *N*, Search space *S*, Number of iterations *I*, Maximum Number of Parameters per architecture *Max*_*Params*_, Population *P*, Fitness score *F*
3: Initialize: *P*←∅
4: for *i* = 1 to *N* do
5: Create individual *I*_*i*_
6: Profile *I*_*i*_ in terms of the ZS-NAS proxies
7: Append *I*_*i*_ to the population *P*
8: end for
9: for *j* = 1 to *I* do
10: Select a random individual *I*_*k*_ from *P*, where *k* = 1, …, *N*
11: Apply Mutation to *I*_*k*_, creating the individual *I*_*k*+1_
12: if *Params*(*I*_*k*_)>*Max*_*Params*_ then
13: goto line 9
14: else
15: Append *I*_*k*+1_ to *P*
16: Calculate the fitness score *F* for *P*
17: Remove the individual with the lowest *F* from *P*
18: end if
19: end for
20: -——————— Second Stage: Fine-Tuning ————————
21: Choose the five individuals *I*_*k*_ with the highest fitness scores and perform training for 100 epochs each
22: Output: The individual *I* with the highest mAP as the architecture output

#### 3.5.3 Training procedure

The same set of hyperparameters will be applied consistently across both datasets for the testbed and the final performance analysis, in line with the procedures outlined in ReYOLOv8 (Silva et al., 2025), Section 3.5.2, and Algorithm 1. The PeDRo dataset (Boretti et al., 2023) was chosen to build the testbed and run stage two of Algorithm 1 due to its small size, while Prophesee's GEN1 (De Tournemire et al., 2020) was selected due to its relevance in the event-based domain as well as because it is a dataset more complex than PeDRo, which should be useful to validate the generalization of the proposed method. All runs involving the PEDRo dataset, as well as the executions of the Chimera-NAS algorithm, were performed on a V100 GPU. In contrast, the runs for the GEN1 dataset and for some larger models in PeDRo were conducted on an A100 GPU. Additional details can be found in Table 3.

This study's training hyperparameters and procedures were primarily adapted from ReYOLOv8 (Silva et al., 2025) and YOLOv8 (Jocher et al., 2023), with minor modifications to batch sizes and learning rates. Table 3 summarizes the hyperparameters used for all runs on PEDRo and GEN1. *LR*0 denotes the initial learning rate, while *LRf* signifies the final learning rate by a linear learning rate schedule. The models were optimized using Stochastic Gradient Descent (SGD) with a momentum of 0.937. Simple grid searches were adopted for the hyperparameters that differ from the literature.

Regarding data augmentation, *HFLlip* refers to horizontal flipping, while *Zoom-Out* was applied with ratios ranging from 1.2 to 1.0. A warmup period of 3 epochs was implemented, featuring a learning rate bias of 0.1 and a warmup momentum of 0.8. The loss functions maintained the same parameters from YOLOv8 (Jocher et al., 2023) alongside the confidence thresholds and non-maximum suppression parameters. The data encoding process over PeDRo was taken by adopting time-windows of 40 ms, while the GEN1 utilized 50 ms for this same purpose, adopting the guidelines from ReYOLOv8 (Silva et al., 2025). Considering the variabilities introduced by the Selective Scan operation (Gu and Dao, 2023), all the models containing Mamba blocks were trained three times, and the average result was taken as final.

## 4 Results

According to the procedures described in Section 3, the first step involved was the training of all the models from the Chimera testbed, as detailed in Sections 3.5.2 and 3.5.3. Next, we analyzed the behavior of various event encodings across different networks. To define the Fitness score used in the search described in Section 3.5.1, we examined the correlations and ordering errors between the proxies introduced in Section 3.4 and the Chimera testbed, selecting the most appropriate data encoding for this purpose. An ablation study was then performed to assess the effectiveness of the linear combination of these proxies in generating high-quality architectures during the Chimera-NAS search, as well as to evaluate the significance of different components within the design space. Finally, we compared the results of our approach with those reported in the literature and presented the computational overhead of the entire process.

### 4.1 Analysis of the event encodings and ZS-NAS proxies

**Figure 5:**
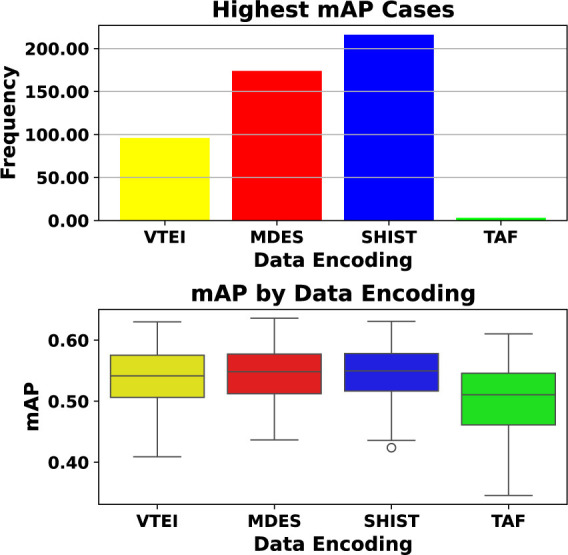
Bar chart and box plot comparing data encodings VTEI, MDES, SHIST, and TAF. The bar chart above shows SHIST with the highest frequency, while the box plot below shows median mAP values around 0.5 across encodings. Different colors represent different encodings. Analysis of testbed and data encodings for the test-set of the PeDRo dataset. Among the combinations of architectures and event encodings, SHIST and MDES consistently demonstrated superior performance in more cases. However, when evaluating the range of mAP values, a similar distribution was observed for VTEI, MDES, and SHIST. Given the highest likelihood of achieving better results, SHIST was selected as the event encoding for the Chimera framework.

The analysis presented in Figure 5 compares various architectures using different data encodings for the Chimera testbed. The upper section of the figure illustrates that the choice of data encoding and architecture should not be made in isolation, as they are interconnected and significantly influence the outcomes. No single encoding guarantees the best performance across all scenarios. Although SHIST and MDES demonstrate superior performance across a greater number of architectures, the lower section of Figure 5 reveals that the distribution of mean Average Precision (mAP) values for SHIST, MDES, and VTEI is quite similar. A notable limitation of the analysis performed here is that the proxies used are data-independent, meaning they cannot distinguish between real event data and random input tensors. It seems that due to the fact of being denser, i.e., retaining more events and not only the last information, as seen in Section 3.1, makes them able to present a more stable performance across different scenarios. As a result, the further analysis was focused on SHIST.

**Figure 6:**
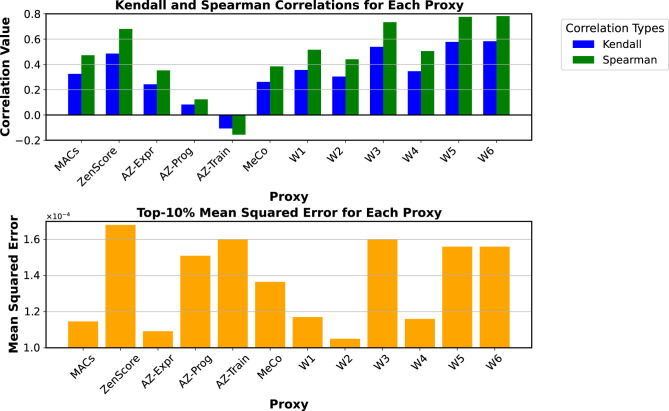
Top graph displays bar plots for Kendall and Spearman correlations across proxies, with Kendall in blue and Spearman in green. Bottom graph shows top-ten-percent mean squared error as orange bars for each proxy, with values ranging from 1.0 to 1.6 × 10^−4^. Proxies include MACs, ZenScore, AZ-Expr, among others. Correlations and Top-10% Mean Squared Errors of the proxies utilized in this work for the SHIST event encoding of the Chimera testbed. The proxies *W*1 to *W*6 represent various configurations of the weight vector **W** from Equation 9. Considering the trade-off between correlation and the MSE, *W*1 was selected as the objective function for the Chimera framework.

To evaluate the efficiency of Zero-Shot proxies for the current application, the testbed rankings were examined using Kendall's and Spearman's correlations for SHIST. The findings are presented in Figure 6. Typically, Neural Architecture Search (NAS) involves primitives that share inherent similarities, such as convolutions with varied branches or alternative operations. However, due to this study's diversity of design paradigms, the Mean Squared Error (MSE) for the top 10% models sorted by each proxy was incorporated into the analysis.

The upper section of the figure reveals that none of the proxies achieve a high Kendall tau correlation, with *ZenScore* performing the best at nearly 50%, followed by MACs, MeCo, and *AZ*^*expr*^. However, when examining the mean squared error (MSE) of the top 10% in the lower section of the same figure, *AZ*^*expr*^ exhibits the lowest error, followed by MeCo and the number of MACs. Despite having a higher correlation than MeCo and *AZ*^*expr*^, *ZenScore* incurs an error 1.67 times greater than these alternatives. While *ZenScore* effectively sorts diverse architectures across the entire testbed, it struggles to identify the top performers. Consequently, there is a complementary relationship between correlation and mean error metrics. In this context, mean squared error becomes critical, highlighting results with minimal errors for top choices.

Figure 6 also presents other proxies derived from linear combinations of the analyzed proxies. The weights for these combinations, detailed in Table 4, were determined using Regression Trees applied to various combinations of proxies to predict the mAP. Due to their underperformance in correlation and MSE metrics, *AZ*^*prog*^ and *AZ*^*train*^ were excluded from this step. The figure indicates that all combinations involving the *ZenScore*—specifically, *W*3, *W*5, and *W*6—demonstrate improvements in Kendall and Spearman correlations, though they yield higher MSE values than other alternatives. Among the remaining combinations, *W*1 achieved the lowest error and correlations. In contrast, *W*1 and *W*4 displayed similar error values, with a slight advantage for *W*1 in correlation. Consequently, considering *W*1 as one of the most balanced solutions in correlation and MSE, it was decided to adopt it as the Fitness Score for Equation 9 and Algorithm 1.

**Table 4:** Analysis of different W vectors for Equation (9).

**W vectors**	**ZS-NAS proxies weights**			
**(Equation 9)**	**ZenScore**	**MAC**	**AZ-Expr**	**MeCo**
**W1**	0.00	0.40	0.00	0.60
**W2**	0.00	0.60	0.40	0.00
**W3**	0.65	0.35	0.00	0.00
**W4**	0.00	0.32	0.18	0.50
**W5**	0.60	0.22	0.00	0.18
**W6**	0.58	0.19	0.06	0.17

The weights were obtained by running Regression Trees over the testbed with different combinations of them.

### 4.2 Ablation studies

#### 4.2.1 Influence of the weight multipliers

To evaluate the impact of the weight multipliers derived in the previous section, we conducted an analysis using the weights listed in Table 4. For the baseline configuration ***W*****1**, perturbations of 0.05 were applied to both the MACs and MeCo scores to assess the sensitivity of the search process to these values. Algorithm 1 was then executed with the different weight settings shown in Table 5, considering five parameter constraints ranging from 3 M to 15 M. The table presents the top-performing architectures from each configuration, evaluated on the PeDRo dataset.

**Table 5:** Comparison of the output of the Algorithm 1, with respect to the PeDRo's dataset mAP, when Equation 9 is implemented adopting different weights for MeCo and MACs, considering the baseline W1 from Table 4, with values disturbed around a range of 0.05.

***MAX*_PARAMS_**	**MeCo=0.55 MACs=0.45**	**MeCo=0.60 MACs=0.40**	**MeCo=0.65 MACs=0.35**
**3M**	**63.0**	62.6	62.1
**5M**	64.8	65.1	**65.7**
**10M**	66.1	**66.5**	**66.5**
**15M**	67.4	67.3	**67.5**

Bold values indicate highest PeDRo mAP per parameter constraint.

From this analysis, it is evident that, on average, there are only minor differences among the various weight settings. However, when the importance of MeCo is slightly increased relative to the baseline *W*1, modest improvements can be observed. It is unsurprising that the *W*1 value obtained via regression is not necessarily optimal, as the Chimera testbed represents only a small subset of the entire design space. Nonetheless, these small perturbations suggest that the regression-derived solution is reasonably close to an optimal region.

#### 4.2.2 Influence of different blocks on the design space

Table 6 presents a comparison of various Chimera Search runs, each omitting a different component from the design space. For every scenario, the procedure described in Section 3.5 was repeated, removing one element at a time. Experiments were constrained to a maximum of 10M parameters, covering a substantial portion of the design space. The mAP values in Table 6 represent the best-performing architecture after training the top five candidates from the ZS-NAS process, using the training protocol detailed in Section 3.5.3.

**Table 6:** Comparison of the mAPs for PeDRo and GEN1 when different parts of the design space are removed, considering a maximum number of parameters of 10 M, the best of the top-5 models, a population of 100 individuals, and 1,000 iterations.

**Design Space**	**C2f**	**Mamba**	**WaveMLP**	**MaxViT**	**mAP PeDRo**	**mAP GEN1**
Full Design Space	x	x	x	x	66.5	**50.4**
No C2f		x	x	x	62.2	43.2
No Mamba	x		x	x	66.9	50.2
No WaveMLP	x	x		x	66.9	49.9
No MaxViT	x	x	x		**67.0**	50.0

Bold values indicate best mAP.

Analysis of the results reveals that the C2f blocks are the most critical components in the library. When C2f blocks are included, the difference between the lowest and highest mAP on the PeDRo dataset is approximately 0.8%. In contrast, excluding C2f blocks leads to a performance drop of 7.7%. This can be attributed to the fact that the framework is built on ReYOLOv8, which is already optimized for such blocks. Since no additional hyperparameter search was performed, it is expected that the best models are biased toward architectures containing C2f blocks.

Comparing the full design space with the reduced alternatives, we observe that, for the PeDRo dataset, reducing the number of blocks yields an improvement of 0.8%. However, for GEN1-a larger and more complex dataset—retaining the full design space results in a mAP that is 0.7% higher than the alternatives. This suggests that, while a smaller design space may better overfit smaller datasets, a comprehensive search across the entire design space is preferable for achieving better generalization.

### 4.3 Search results

Table 7 presents the top five architectures identified during the first stage of the Chimera search, as discussed in Section 4.3. It also displays the mean Average Precision (mAP) scores for PeDRo obtained in the second stage. The architectures selected for output are highlighted in bold.

**Table 7:** Top-5 architectures resulting from the 1st stage of Algorithm 1.

**Architecture details**	**1**	**2**	**3**	**4**	**5**
**ZS-NAS ranking**, *MAX*_PARAMS_ **= 3M**					
**mAP (PeDRo)**	61.87	60.86	60.31	**62.06**	61.24
**Chimera Layer 1**	c2f	c2f	c2f	**c2f**	c2f
**Chimera Layer 2**	c2f	c2f	c2f	**c2f**	c2f
**Chimera Layer 3**	c2f	c2f	c2f	**c2f**	c2f
**Chimera Layer 4**	WaveMLP	WaveMLP	MaxViT	**WaveMLP**	WaveMLP
**ZS-NAS ranking**, *MAX*_PARAMS_ **= 5M**					
**mAP (PeDRo)**	**65.73**	63.39	64.54	63.29	64.99
**Chimera Layer 1**	**c2f**	c2f	c2f	c2f	c2f
**Chimera Layer 2**	**c2f**	c2f	c2f	c2f	c2f
**Chimera Layer 3**	**c2f**	c2f	c2f	WaveMLP	c2f
**Chimera Layer 4**	**MaxViT**	MaxViT	c2f	c2f	MaxViT
**ZS-NAS ranking**, *MAX*_PARAMS_ **= 10M**					
**mAP (PeDRo)**	64.52	**66.5**	65.25	64.35	64.75
**Chimera Layer 1**	C2f	**c2f**	C2f	C2f	C2f
**Chimera Layer 2**	C2f	**c2f**	C2f	C2f	C2f
**Chimera Layer 3**	Mamba	**WaveMLP**	Mamba	Mamba	Mamba
**Chimera Layer 4**	C2f	**c2f**	C2f	C2f	C2f
**ZS-NAS ranking**, *MAX*_PARAMS_ **= 15M**					
**mAP (PeDRo)**	65.44	67.36	66.44	**67.5**	65.92
**Chimera Layer 1**	c2f	c2f	c2f	**c2f**	c2f
**Chimera Layer 2**	c2f	c2f	c2f	**c2f**	c2f
**Chimera Layer 3**	c2f	c2f	c2f	**WaveMLP**	WaveMLP
**Chimera Layer 4**	MaxViT	c2f	MaxViT	**c2f**	c2f

Bold values indicate selected configuration.

Notably, there are variations among the top five architectures. These discrepancies arise from the proxies' inaccuracies in predicting optimal performance, which underscores the necessity for the second stage of the Chimera algorithm detailed in Algorithm 1. Table 8 shows more details regarding the Chimera models, such as the number of channels and repeats.

**Table 8:** Detailed parameters from the backbones of the Chimera models.

**Block**	**Processing unit**	**Input channel**	**Output channel**	**Repeats**
**Chimera-n0**				
STEM	Conv2D	10	24	1
Chimera Layer 1	C2f	24	40	3
Chimera Layer 2	C2f	40	56	3
Chimera Layer 3	C2f	56	72	3
Chimera Layer 4	WaveMLP	72	80	2
**Chimera-n**				
STEM	Conv2D	10	32	1
Chimera Layer 1	C2f	32	56	3
Chimera Layer 2	C2f	56	104	3
Chimera Layer 3	C2f	104	112	3
Chimera Layer 4	MaxVit	112	144	1
**Chimera-s**				
STEM	Conv2D	10	40	1
Chimera Layer 1	C2f	40	72	3
Chimera Layer 2	C2f	72	112	3
Chimera Layer 3	WaveMLP	112	128	1
Chimera Layer 4	C2f	128	216	2
**Chimera-m**				
STEM	Conv2D	10	48	1
Chimera Layer 1	C2f	48	96	3
Chimera Layer 2	C2f	96	160	3
Chimera Layer 3	WaveMLP	160	160	2
Chimera Layer 4	C2f	160	160	1

Building on the analysis from the previous sections, Algorithm 1 was executed using the weight vector **W** obtained from Section 4.1. Different architectures were obtained by running the search in four different cases, where *MAX*_Params_ was set to 3 M, 5 M, 10 M, and 15 M, designated as Chimera-n0, Chimera-n, Chimera-s, and Chimera-m, respectively. For the first three cases, a population size of 100 and 1,000 iterations was utilized, while for the 15 M case, both numbers were doubled.

From the previous Tables, it can be seen that the search mechanism favored configurations dominated by C2f blocks. This preference aligns with the successful outcomes of ReYOLOv8 (Silva et al., 2025), especially considering that no additional hyperparameter optimization was performed. Additionally, WaveMLPs and MaxViT were utilized exclusively in Chimera Layers 3 and 4, which are richer in features despite having lower resolutions. This allocation represents a more effective use of these blocks, as they are generally more efficient for global context information extraction than convolutions. However, they are not the optimal choice for spatial feature extraction. In contrast, the higher-resolution layers were populated solely by C2f blocks, which are expected to be more efficient for extracting spatial information at these resolutions than the alternatives.

### 4.4 Comparison with the state-of-the-art

Table 9 presents the results of the Chimera architectures on the PeDRo dataset (Fan et al., 2025). When comparing the Nano and Small Scales models, it is evident that the Chimera models excel. Specifically, Chimera-n outperformed ReYOLOv8n by 2.81% with only a negligible increase in the number of parameters. Additionally, a +1.0 increase in mAP was observed when comparing Chimera-s to ReYOLOv8s. Notably, Chimera-m achieved 97.6% of the mAP reported for ReYOLOv8m while requiring 1.3 times fewer parameters. It is important to mention that ReYOLOv8 was trained using VTEI encoding and incorporated a data augmentation technique tailored for this encoding, which appears particularly effective for this dataset (Silva et al., 2025).

**Table 9:** Comparison of the Chimera models with other models in literature for the PeDRo dataset.

**Scale**	**Model**	**Network**	**Parameters**	**mAP**
Nano	Chimera-n0 (this work)	Hybrid + RNN	2.5M	62.1
		ReYOLOv8n Silva et al. (2025)	CNN + RNN	4.7M	63.9
		Chimera-n (this work)	**Hybrid + RNN**	**4.9M**	**65.7**
Small	ReYOLOv8s Silva et al. (2025)	CNN + RNN	8.4M	65.5
		Chimera-s (this work)	**Hybrid + RNN**	**10.0M**	**66.5**
>10M	Chimera-m (this work)	Hybrid + RNN	13.8M	67.5
		ReYOLOv8m Silva et al. (2025)	**CNN + RNN**	**18.1M**	**69.1**
		YOLOv8x Boretti et al. (2023)	CNN	68.2M	58.6

Bold values indicate highest mAP per scale.

To evaluate the generalization capability of the Chimera models, training was conducted on Prophesee's GEN1 dataset, with corresponding results detailed in Table 10. The runtimes are based on a GTX 1080 Ti GPU, similar to the setup used in ReYOLOv8 (Silva et al., 2025). Across all model scales, the Chimera models consistently outperformed their counterparts. Specifically, Chimera-n demonstrated a 6.7% performance improvement over ReYOLOv8n while maintaining a similar parameter count. Meanwhile, Chimera-n0 showed a 2.3% improvement while utilizing nearly half the model size of ReYOLOv8n. On bigger scales, Chimera-s not only surpassed all the similarly scaled models but also matched the performance of SMamba, the previous state-of-the-art model, requiring 1.61 × fewer parameters and being 2.1 times faster. Finally, Chimera-m established a new testbed for the GEN1 dataset, exceeding the previous best score by 1% while also reducing the model size by 14.3% and achieving a 1.35 × speed-up.

**Table 10:** Comparison with the state-of-the-art for the Prophesee's GEN1 dataset for different scales.

**Scale**	**Model**	**Network**	**Parameters**	**GFLOPs**	**Runtime**	**mAP**
Nano	Chimera-n0 (this work)	Hybrid + RNN	2.5 M	1.0	10.6 ms	47.4
		RVT-T (Gehrig and Scaramuzza 2023)	Transformer + RNN	4.4 M	0.9	9.4 ms	44.1
		ReYOLOv8n Silva et al. (2025)	CNN + RNN	4.7 M	1.2	9.2 ms	46.3
		Chimera-n (this work)	Hybrid + RNN	4.9 M	2.3	10.9 ms	**49.4**
Small	EMS-YOLO Su et al. (2023)	SNN	6.2 M	-	-	26.7
		Spiking DenseNet Cordone et al. (2022)	SNN	8.2 M	-	-	18.9
		ReYOLOv8s Silva et al. (2025)	CNN + RNN	8.4 M	2.3	10.4 ms	48.3
		RVT-S Gehrig and Scaramuzza (2023)	Transformer + RNN	9.9 M	1.8	9.5 ms	46.5
		Chimera-s (this work)	Hybrid + RNN	10.0 M	3.5	11.4 ms	**50.4**
>10 M	SFOD Fan et al. (2024)	SNN	11.9 M	-	-	32.1
		Chimera-m (this work)	Hybrid + RNN	13.8 M	6.2	17.8 ms	**50.9**
		SpikeSSD-S Fan et al. (2025)	SNN	13.9 M	-	-	39.0
		SMamba Yang et al. (2025)	SSM + RNN	16.1 M	2.4	24.0 ms	50.4
		ReYOLOv8m Silva et al. (2025)	CNN + RNN	18.1 M	4.7	12.3 ms	49.5
		S5-ViT-B Zubic et al. (2024)	Transformer + SSM	18.2 M	>3.1	9.4 ms	47.7
		RVT-B (Gehrig and Scaramuzza 2023)	Transformer + RNN	18.5 M	3.5	10.2 ms	47.2
		SAST-CB Peng et al. (2024)	Transformer + RNN	18.9 M	2.4	22.7 ms	48.2
		SpikeSSD-L Fan et al. (2025)	SNN	19.0 M	-	-	40.8
		GET-T Peng et al. (2023b)	Transformer + RNN	21.9 M	3.6	16.8 ms	47.9
		RED Perot et al. (2020)	CNN + RNN	24.1 M	6.0	16.7 ms	40.0
		EAS-SNN Wang et al. (2024)	SNN	25.3 M	-	-	37.5
		ERGO-12 Zubić et al. (2023)	Transformer	59.6 M	50.8	69.9 ms	50.4
		ASTMNet Li et al. (2022a)	CNN + RNN	>100 M	20.3	35.6 ms	46.7

The GFLOPs are related to the backbones. Bold values indicate highest mAP per scale.

### 4.5 Chimera-NAS runtime

Table 11 presents the runtime for the various steps involved in Chimera-NAS. This analysis considered a population of 50 individuals, 1,000 iterations, and a maximum parameter count of 5 M per model. Stage 1 implements the ZS-NAS step from Algorithm 1. This stage was conducted on an Ubuntu OS with 265 GB of RAM, an Intel Xeon Gold @2.10 GHz × 104 processor, and a Quadro RTX 4000 GPU. Under this setup, Chimera-NAS evaluated 1,050 models in 1.32 h, which is 2.64 times faster than a complete training session of 100 epochs on the PEDRo dataset using an NVIDIA V100 GPU, as shown in the table.

**Table 11:** Analysis of the runtimes involved in the Chimera framework.

**Item**	**Runtime**
Stage 1 (ZS-NAS)	1.32 h
Training - 100 epochs (v100 GPU)	3.48 h
Stage 2 (Train top-5 from ZS-NAS)	17.4 h

## 5 Conclusions

This work presents a two-stage NAS approach specifically aimed at Event-Based Object Detection. Rather than merely exploring variations of specific blocks, the architecture search focused on combining blocks from various paradigms within the literature to construct more robust architectures. The resulting framework, named Chimera, employs proxies to evaluate architecture performance on test sets without requiring extensive training, enabling the examination of over 1,000 structures within a few hours.

For benchmarking and conducting the architecture search, we utilized the PeDRo dataset. From this benchmarking, it was possible to analyze the interdependence between the choice of event encoding and the underlying architecture regarding final performance, underscoring the importance of co-designing these elements. Different Zero-Shot NAS proxies were analyzed in terms of correlation and error relative to benchmark performance. Not all proxies provided optimal outcomes in this multi-paradigm scenario, necessitating the use of Regression Trees to identify the best combinations of them for the search mechanism.

Subsequently, models with parameter scales ranging from 3 M to 15 M were generated through this search. The final models not only achieved competitive performance on the PeDRo dataset but also demonstrated strong generalization to the larger and more complex Prophesee's GEN1 dataset. For the GEN1 dataset, one of the models, designated Chimera-s, exhibited state-of-the-art mean Average Precision (mAP) while reducing the number of parameters by 1.6 × and achieving a speed-up of 2.1 × . Additionally, Chimera-m established a new benchmark for this dataset, surpassing the previous best score by 1% while reducing the model size by 14.3% and achieving a speed-up of 1.35 × .

## 6 Future works

Future work will focus on expanding the exploration of additional blocks and alternative types of memory cells, including State Space Models. We also intend to utilize larger datasets, particularly Prophesee's 1 MegaPixel dataset and eTraM. Additionally, we will investigate incorporating hyperparameter optimization into the Chimera framework, which could contribute to identifying more diverse architectures with enhanced performance.

## Data Availability

The original contributions presented in the study are included in the article/supplementary material, further inquiries can be directed to the corresponding author.
